# Bioethics and medicine

**DOI:** 10.3325/cmj.2013.54.1

**Published:** 2013-02

**Authors:** Gordana Pelčić

**Affiliations:** Department of Social Sciences and Medical Humanities, University of Rijeka School of Medicine and Health Care Center of Primorsko-Goranska County, Rijeka, Croatia

Dedicated to the memory of Prof. Ivan Šegota, PhD

International Society for Clinical Bioethics (ISCB) together with the Department of Social Sciences and Medical Humanities, University of Rijeka School of Medicine organized the 9th ISCB Conference in September 2012 dedicated to the memory of Prof. Ivan Šegota, PhD (1).

The story of bioethics and medicine at the University of Rijeka School of Medicine started several decades ago, with professor Šegota’s and professor Volarić’s idea to design an interdisciplinary course of Medical Ethics and Deontology (2). The idea was revived in 1993 when Prof. Šegota visited respectable bioethical centers such as Hasting center, Kennedy Institute of Ethics, etc. The acquired knowledge and ideas were included in the book New Medical Ethics (Bioethics) published in 1994 (3).

Prof. Šegota always emphasized the effort that his colleagues, students, and assistants invested into the development of bioethics and medicine in Croatia. Prof. Šegota’s idea was to open the “bioethical doors” to the medical professionals. During the years, the Department of Social Sciences in Rijeka (today the Department of Social Sciences and Medical Humanities) assembled many young scientists and reputable experts devoted to the idea of bioethics. The first PhD thesis in bioethics in Croatia was written in Rijeka by Prof. Aleksandra Frković, entitled The Informed Consent in Theory and Practice of Clinical Bioethics. Later on she published two remarkable books on bioethics and clinical practice (4,5). Bioethical education was investigated in the PhD thesis of Nada Gosić and was later elaborated in the book Bioethics Education (6). The palliative medicine and hospices, also bioethical subjects, were discussed at the Bioethical Round Table in 2005 (7), and the following year this topic become the field of interest of our colleague Morana Brkljačić (8). Emerging interest in bioethics and medicine contributed to the foundation of the first Croatian hospice opened in Rijeka in the beginning of 2013.

Experts from the Department of Social Science and Medical Humanities have also dealt with many other bioethical issues, such as transplantation medicine (9), bioethical consultation first investigated by IS Bilajac (10), the rights of the patient and communication issues (11,12), communication with deaf patients (13), European bioethics and European roots of bioethics (14,15), and the language of medicine (16).

The collaboration and communication with foreign bioethical experts started in 1990, and in 1996 many respectable bioethical experts such as Peter Singer, Tom L Beauchamp, Ruth Chadwick, Warren T Reich, Robert M. Veatch, Rihito Kimura, Hans-Martin Sass, and Darryl Macer published their articles in the issue of *Društvena Istraživanja* entitled “New Medical Ethics” (17).

The close collaboration with the eminent world bioethicists resulted in publishing their work in the Croatian translation (18,19). Especially important was the publication of Potter’s Bioethics: Bridge to the Future (18). Potter’s bioethical bridge is the bridge to the future but it can also be the bridge between bioethics and other fields of science or life. Every person, scientist, or medical professional can develop their own bridge to bioethics. Prof. Luka Tomašević in the book From New Medical Ethics to the Integrative Bioethics emphasized the essence of bioethics as love of life (20). Many more Croatian scientists developed bridges between their field of interest and bioethics.

The bridge between bioethics and medicine is essential and natural. Each of us decides which position and role he or she will take. The dream of Prof. Šegota was an active role of medical professionals in the field of bioethics so that they can contribute to the bioethical issues from their perspective. Education of medical professionals in bioethics might help to initiate preventive bioethical action in everyday medicine.

Many actions have been taken in the past decades with the goal to make that dream come true and have been supported by the Rijeka University, the City, and the County. One of the actions was the foundation of ISCB, which gathered bioethicists from different scientific fields and different parts of the world with the purpose of creating a connection between medicine and bioethics. The need for the bridge between these two disciplines should constantly be emphasized, with the hope that the bridge will become firm and a part of our everyday life. This issue of the *Croatian Medical Journal*, dedicated to bioethics and medicine, invites researchers to the study of many diverse bioethical issues. Bioethics requires courage to speak out about different issues in our own field regardless of the social or political situation. The history of bioethical movement and its connection with medicine in Croatia justifies this possibility and contributes to its future realization.
